# White paper on peanut allergy – part 1: Epidemiology, burden of disease, health economic aspects

**DOI:** 10.1007/s40629-021-00189-z

**Published:** 2021-09-28

**Authors:** Lars Lange, Ludger Klimek, Kirsten Beyer, Katharina Blümchen, Natalija Novak, Eckard Hamelmann, Andrea Bauer, Hans Merk, Uta Rabe, Kirsten Jung, Wolfgang Schlenter, Johannes Ring, Adam Chaker, Wolfgang Wehrmann, Sven Becker, Norbert Mülleneisen, Katja Nemat, Wolfgang Czech, Holger Wrede, Randolf Brehler, Thomas Fuchs, Thilo Jakob, Tobias Ankermann, Sebastian M. Schmidt, Michael Gerstlauer, Torsten Zuberbier, Thomas Spindler, Christian Vogelberg

**Affiliations:** 1 Department of Pediatrics, St. Marien-Hospital, GFO Clinics Bonn, Bonn, Germany; 2 Center for Rhinology and Allergology Wiesbaden, Wiesbaden, Germany; 3 Department of Pediatrics m.S. Pneumology, Immunology and Intensive Care Medicine, Charité—Universitätsmedizin Berlin, Berlin, Germany; 4 Center of Pediatric and Adolescent Medicine, Focus on Allergology, Pneumology and Cystic Fibrosis, University Hospital Frankfurt, Goethe University Frankfurt, Frankfurt a. M., Germany; 5 Clinic and Polyclinic for Dermatology and Allergology, University Hospital Bonn, Bonn, Germany; 6 Pediatric and Adolescent Medicine, Bethel Children’s Center, OWL University Hospital of Bielefeld University, Bielefeld, Germany; 7 Clinic and Polyclinic for Dermatology, University AllergyCenter, University Hospital Carl Gustav Carus, Dresden University of Technology, Dresden, Germany; 8 Department of Dermatology & Allergology, RWTH Aachen, Aachen, Germany; 9 Clinic for Allergology, Johanniter-Krankenhaus im Fläming Treuenbrietzen GmbH, Treuenbrietzen, Germany; 10 Practice for Dermatology, Immunology and Allergology, Erfurt, Germany; 11 Medical Association of German Allergists, Dreieich, Germany; 12 Skin and Laser Center at the Opera, Munich, Germany; 13 Department of Otolaryngology, Klinikum rechts der Isar, Technische Universität München, Munich, Germany; 14 Center for Allergy and Environment (ZAUM), Klinikum rechts der Isar, Technische Universität München, Munich, Germany; 15 Wehrmann Dermatological Group Practice, Münster, Germany; 16 Department of Otolaryngology, University of Tübingen, Tübingen, Germany; 17 Asthma and Allergy Center Leverkusen, Leverkusen, Germany; 18 Pediatric Pneumology/Allergology Practice, Kinderzentrum Dresden (Kid), Dresden, Germany; 19 Practice and clinic for allergology/dermatology, Schwarzwald-Baar Klinikum, Villingen-Schwenningen, Germany; 20 Practice and clinic for allergology/ear, nose and throat specialist, Herford, Germany; 21 Clinic for Skin Diseases, Outpatient Clinic for Allergology, Occupational Dermatology and Environmental Medicine, Münster University Hospital, Münster, Germany; 22 Department of Dermatology, Venereology and Allergology, University Hospital, Georg-August-University, Göttingen, Germany; 23 rd Clinic for Dermatology and Allergology University Hospital Giessen, UKGM Justus Liebig University Giessen, Giessen, Germany; 24 th Clinic for Pediatric and Adolescent Medicine, Pneumology, Allergology, Neonatology, Intensive Care Medicine, Infectiology, University Hospital Schleswig-Holstein, Kiel, Germany; 25 th Center for Pediatric and Adolescent Medicine, Clinic and Polyclinic for Pediatric and Adolescent Medicine, Greifswald University Medical Center, Greifswald, Germany; 26 pediatric pneumologist/pediatric allergologist, II. clinic for children and adolescents, University Hospital Augsburg, Augsburg, Germany; 27 Clinic for Dermatology, Venereology and Allergology, Charité—Universitätsmedizin Berlin, Berlin, Germany; 28 Department of Pediatrics and Adolescent Medicine, Pediatric Pneumology, Allergology, Sports Medicine, Hochgebirgsklinik Davos, Davos-Wolfgang, Switzerland; 29 TU Dresden/UKDD, Pediatric Department, University Hospital Dresden, Dresden, Germany; 30 Department of Pediatric Pneumology/Allergology, Clinic and Polyclinic for Pediatrics and Adolescent Medicine, University Hospital Carl Gustav Carus, Fetscher Street 74, 01307 Dresden, Germany

**Keywords:** Food allergy, Anaphylaxis, Oral immunotherapy, COVID-19, Children

## Abstract

Peanuts are Leguminosae, commonly known as the legume or pea family, and peanut allergy is among the most common food allergies and the most common cause of fatal food reactions and anaphylaxis.

The prevalence of peanut allergy increased 3.5-fold over the past two decades reaching 1.4–2% in Europe and the United States. The reasons for this increase in prevalence are likely multifaceted. Sensitization via the skin appears to be associated with the development of peanut allergy and atopic eczema in infancy is associated with a high risk of developing peanut allergy.

Until recently, the only possible management strategy for peanut allergy was strict allergen avoidance and emergency treatment including adrenaline auto-injector in cases of accidental exposure and reaction.

This paper discusses the various factors that impact the risks of peanut allergy and the burden of self-management on peanut-allergic children and their caregivers.

## Introduction

Peanuts belong to the botanical family Leguminosae, commonly known as the legume or pea family [1]. Peanut allergy is among the most common food allergies and now the most common cause of fatal food reactions [1, 2]. Peanuts are one of the food allergens most commonly associated with anaphylaxis, a sudden and potentially deadly allergic reaction that requires immediate attention and treatment. Although food allergy-related fatalities are rare, peanut allergy accounts for most of them, even in individuals with a history of mild reactions, making prediction difficult [3].

Most food allergies, for example, those to egg and milk, are often limited to infancy and are usually “outgrown” in childhood. This is only the case in less than 20% of children with peanut allergy [4–7]. Children with a low initial sensitization, i.e., a low initial peanut-specific Immunoglobulin E (IgE) level, and those with only cutaneous symptoms without other accompanying symptoms, are more likely to outgrow their peanut allergy. Outgrown peanut allergy also coincides with lower rates of atopic eczema and other comorbidities generally seen in peanut-allergic patients [7].

Until recently, the only possible management strategy for peanut allergy was limited to the combination of strict allergen avoidance along with an action plan, including having an adrenaline auto-injector (AAI) on hand in case of accidental exposure and reaction to peanut, which is sometimes referred to as an “avoidance management strategy.” This white paper discusses various factors related to the impact of the risks of peanut allergy and the burden of self-management on peanut-allergic children and their caregivers.

## Epidemiology

The prevalence of peanut allergy in the United States has been reported to have increased 3.5-fold over the past two decades, from 0.4% in 1997 to 0.8% in 2002 and 1.4% in 2008 [8–10]. Currently, 1–2% of children are affected in the Western world [11–13]. Although the trend in increased prevalence of peanut allergy is seen in most regions, it is also important to note that the variability of estimates is in part due to the different diagnostic methods, the age of the cohorts, and the populations studied [11–13]. The reasons for the increase in prevalence of peanut allergy are not known and are likely multifaceted; however, sensitization via the skin appears to be associated with the later development of peanut allergy [14] and atopic eczema in infancy is associated with a high risk of developing peanut allergy [15]. Several studies have shown that disturbances in cutaneous barrier function—e.g., with lower formation of filaggrin—may promote peanut sensitization [16, 17]. By contrast, early and regular consumption of peanut protein from infancy onward in relevant amounts promotes tolerance development, especially in at-risk children with atopic eczema or other food allergies [18–20].

Peanut and hazelnut allergies frequently occur at preschool age, in 55% of children by 2 years of age and in 92% by 7 years of age [21]. The later onset of clinical symptoms is usually explained by later first consumption. The development of primary allergy to peanut after previous problem-free consumption is a rarity. Approximately one third of patients are clinically allergic both to peanuts and to tree nuts [21]. In a recent prospective study of cross-allergy in peanut and nut allergic patients by Brough et al., approximately 30% of patients also reacted to cashew, 28% to walnut and pistachio, 22% to hazelnut, and 20% to pecan [22].

## Clinical symptoms and diagnostics

Allergies to peanut have a range of clinical presentations from cutaneous manifestations to life-threatening systemic reactions. Peanut allergy mostly manifests as isolated cutaneous symptoms (94%), or as respiratory tract (42%) and/or gastrointestinal system (33%) symptoms. An allergic response to peanuts usually occurs within minutes of exposure. In one study, 95% of patients reacted within 20 min [23]; in another study, the median onset of a reaction after oral challenge was as late as 55 min [24]. In large cohort studies, approximately one third of patients reacted with the clinical symptoms of anaphylaxis to accidental consumption [25, 26]. Some allergic patients react to very small (milligrams) amounts of peanut protein, but many react only to larger amounts equivalent to more than one peanut kernel [25–30]. In a survey of 669 peanut-allergic participants, the amount of food allergen triggering the accidental reaction was able to be estimated in 238 participants (35.5%). Median estimated eliciting dose in real life was 125 mg (interquartile range: 34–177 mg) of peanut protein [25].

To better assess the different risk profiles, a whole series of peanut molecular antigens (allergen components) have been identified so far (Ara h 1–11; [31–33]). Of these, Ara h 1, 2, 3, and 6 are associated with higher-grade allergic/anaphylactic reactions after peanut protein exposure, and the majority of clinically relevant peanut-allergic patients produce antigen-specific IgE antibodies to these allergens [34–37]. Elevated serum IgE levels for the Ara h 2 component have been shown to be particularly relevant for diagnostics [38, 39]. Specific IgE against Ara h 8, a PR10 protein and Bev 1-homologous allergen, on the other hand, indicates a cross-allergy in the context of an existing birch pollen sensitization, with absent or only mild symptoms on peanut consumption, most likely in the context of an oral allergy syndrome.

Double-blind placebo-controlled oral allergen challenge (DBPCFC) is considered the gold standard for the diagnosis of food allergy, including peanut allergy [40]. However, in daily practice, a combination of a typical history of an allergic reaction and a positive skin prick test or the detection of serum-specific IgE antibodies against peanut, and especially against the peanut Ara h 2 storage protein, often confirms the diagnosis of a clinically relevant peanut allergy.

## Burden of disease and impact on quality of life

The daily burden due to peanut allergy can be substantial [41]. Peanut-allergic children have a poorer quality of life than children with diabetes mellitus [42], mainly due to the potential dangers in the everyday environment and the fear of fatal anaphylaxis [42]. A recent Europe-wide study shows that peanut allergy has a day-to-day impact on more than 80% of affected children and their parents/caregivers. In comparison, nearly 40% live with a high or extremely high level of stress, and a similar proportion of peanut-allergic individuals reported feeling frequently or very frequently frustrated because of their allergy [43]. In this regard, the processing strategies of families are very different [44]. A study from the United States showed that approximately 40% of patients had a good coping strategy characterized by high competence, with little anxiety and few restrictions in everyday life. Another about 45% of affected families have high competence, but also much fear of reactions and thus moderate limitations [44]. Only approximately10% of families are paralyzed with anxiety. The cause of the fears and quality-of-life limitations is mainly the concern of severe allergic reactions due to accidental ingestion of peanut. In this regard, “trace” peanut, i.e., the unintentional introduction of allergen into processed foods, usually plays a role. Contamination by peanut proteins does occur [45, 46]. In the majority of cases of fatal and near-fatal reactions to peanut, patients were unaware that the foods consumed contained peanut proteins, suggesting that attempts at consistent avoidance are not easily implemented [1, 47]. Parents often do not feel sufficiently understood and supported by the environment [41]. On the other hand, families with an affected child usually have good cohesion, which they perceive as strengthening [41].

The degree of anxiety of the families clearly depends on the given assessment of the situation and recommendations of the physicians in charge [25]. If rigorous allergen avoidance is recommended, the families’ anxiety is greater. Often, these patients and their families avoid eating in restaurants because of the risk of food contamination with peanut, which is not apparent there [42, 48]. Shopping can be time-consuming (due to review of food labels), frustrating, and limited because a great many products are labeled “may contain peanut” even when it seems unlikely that they contain significant amounts [42, 48].

## Socioeconomic impact

The presence of peanut allergy leads to high costs for the healthcare system [49]. On the one hand, peanut allergy itself incurs costs due to prescription of emergency medication or planned and unplanned physician visits. On the other hand, many patients with peanut allergy also suffer from other atopic diseases such as bronchial asthma and atopic eczema, which causes additional high costs. A recent study in the United Kingdom demonstrated that compared to matched control groups (normal and with/without an atopic condition), patients with peanut allergy had a greater number of contacts (per person-year) with primary care providers, inpatient care, prescriptions, outpatient care, and accident and emergency admissions [50]. While many studies examining the socioeconomic impact of peanut allergy have limitations, the overall trend toward increased cost to the healthcare system is apparent.

## Management and therapeutic options

The standard of care to date has been to educate patients, caregivers, and families to avoid peanuts and peanut-containing products and to prescribe emergency medications (injectable intramuscular adrenaline/epinephrine, oral antihistamines, oral steroids, inhaled β2-agonists) to be used as needed [51–53].

To ensure safe use, instructions on the application of emergency medications should be provided with the prescription, and the patient or, in the case of young children, their caregivers should be encouraged to attend AGATE (*Arbeitsgemeinschaft Anaphylaxie Training und Edukation*) anaphylaxis training sessions [54–56] These training sessions explain in detail both the management of allergic reactions and allergen avoidance strategies.

Unfortunately, many families of peanut-allergic children know little about how to avoid food allergens, treat accidental food exposures, and use an epinephrine auto-injector [57]. This is compounded by nearly one third of nut-allergic children being unable to reliably identify the nuts to which they are allergic [58]. Furthermore, peanut avoidance is nearly impossible given that peanut has become a ubiquitous foodstuff, used in many different foods, and labeling may be inadequate or misinterpreted by families and caregivers [53, 59]. It is therefore not surprising that reactions after accidental ingestion are recurrent, especially in school settings [60] and at meals away from home, for example, in restaurants [61].

The risk of accidental exposure to peanut is still high among individuals with peanut allergy. Data on the annual incidence rate of reactions due to accidental exposures vary, likely due to variations in data collection, geographic regions, and time of study. An incidence of 15% has been reported in a group of 567 patients with nut allergy who were referred to an outpatient allergy clinic and followed up annually [62]; an incidence of 55% over 5 years in a cohort of 102 peanut-allergic children [23]; and a rate of 75% over a 14-year period [4]. In a recent pooled analysis of several studies, a rate of approximately 10% adverse reactions per capita per year was calculated [63].

A comprehensive education and management plan that includes verbal and written advice on nut avoidance and treatment of allergic reactions can effectively reduce both the severity and the number of future reactions [57, 62, 64]. In this regard, the AGATE training program is also recommended, as it explains in detail both the management of allergic reactions and allergen avoidance strategies [54, 56]. Moreover, AGATE training courses for caregivers given by patient advocacy groups like the DAAB (German Allergy and Asthma Association) also fulfill this purpose. In addition, caregivers and staff of daycare centers and schools should also be instructed and trained in the management of allergy and the use of the emergency medication.

## European precautionary allergen labeling

The European Union (EU) standardized food labeling regulation governs how packaged food must be labeled and what minimum information must appear on the packaging. The basis for this is the European Food Information Regulation (LMIV; EU) No. 1169/2011, which has applied to allergens since December 13, 2014. The EU regulation applies directly in all EU member states. It can be supplemented by member states national guidelines and regulations in certain cases.

### Allergen labeling regulations on packaged goods

The European Food Information Regulation (LMIV; EU) No. 1169/2011 requires that the 14 most important substances (including peanuts) that can trigger allergies or intolerances be listed on the ingredient label of packaged product. The 14 substances listed in Annex II of the Food Information Regulation (LMIV; EU) No. 1169/2011 are: cereals containing gluten, crustaceans, fish, soybeans, milk, eggs, nuts, celery, mustard, lupine, sesame seeds, mollusks, sulfites, and also peanuts and products derived from peanuts.

These substances must be highlighted in the list of ingredients, e.g., the font style (e.g., bold print) or the background color/shading. The labeling requirement also applies to all allergenic substances and excipients used in production. If there is no list of ingredients, the substances must be indicated with the additional note “contains”, for example, “contains peanuts.”

### Allergen labeling of loose goods

Information on the allergen content of food is also mandatory for unpackaged goods (e.g., at the service counter or in restaurants). This information may be provided in writing, electronically, or orally. In the case of oral information, written documentation must be readily available upon request. This can be done on the basis of the suggestions developed by the associations, e.g., as a leaflet, information sheet, recipe details, or similar. There must be a clear indication of this at the point of sale.

## “Trace identification”

While the labeling of allergen entries deliberately added to a prepared food due to the recipe is required by law, the declaration of unintentional allergen entries (“traces of”) is not required by law. Manufacturers are allowed to decide individually whether or not to include a corresponding note under the list of ingredients. Considering how contamination can occur, it is difficult to produce food that is guaranteed to be free of an allergen. This is possible for some large manufacturers who operate entire plants dedicated to peanut- and nut-free production for this purpose. However, it is not only in the factory that allergens can be transferred through the shared use of a production line. All suppliers and source products must also be checked, and it should be possible to guarantee that no allergen input can have taken place. A study from the United States was able to show that large, internationally active food groups are more likely to have appropriate quality management in place, which reduces the risk of corresponding allergen entries [65].

The term “trace” does not at all mean that only small amounts of the allergen are present as unintentional contamination. In certain products such as mueslis, nut pastries, or chocolates, quantities in the range of whole peanut kernels can also occur as a “trace.”

Since the declaration is voluntary, smaller companies and manufacturers of loose goods in particular decide either not to provide any trace information at all or to provide information on all possible allergens “just to be on the safe side.” An extensive review paper by Brough et al. summarizes the recommendations on the basis of the available data in such a way [66] that in the case of highly sensitive patients, high-risk products in particular should be avoided, since contamination can occur here even without appropriate trace labeling (Table 1). Contamination with peanut protein in other products such as ready meals, on the other hand, occurs only rarely.

**Table 1:** Products with high risk of contamination with peanut allergens (according to [66])

Chocolate and candy bar
Cookies and biscuit
Muesli bar, fruit bar, protein bar
Nut mix, “Nibbles”
Bakery products (cakes, pastries, pies, rarely grain bread)
Confectionery products
Ice cream
Restaurant prepared food, especially Asian cuisine

## Discussion

Peanut allergy is one of the most common food allergies in Western nations and is often a lifelong condition [1, 2]. Allergy to peanut is among the common causes of food-allergy-related anaphylaxis and emergency department admissions [1, 2] The diagnosis of a patient with suspected peanut allergy may include a careful history taking, skin-prick testing, measurement of serum-specific IgE, and, possibly, an oral food challenge, all of which are discussed in Part 2 of this white paper (Blum et al. in this issue of *Allergo Journal*).

To date, management options for peanut allergy combined a strict allergen avoidance along with an action plan with emergency treatment in the case of reaction due to accidental exposure to peanut, as described in detail in the contribution by Reese et al. in Part 4 of this white paper (Reese et al. in this issue of *Allergo Journal*).

Although practical and well-established dietary regimens have been developed for this purpose [67], peanut allergy represents a considerable burden on the lives of affected individuals, their families, and caregivers.

The information received by parents and caregivers can impact strongly on their quality of life, sometimes much more than their actual experiences. Parents obtain information from various sources, often unfiltered from the Internet. The allergists in charge should provide families with verified information and give a risk assessment based on the known individual influencing factors (such as the known individual reaction amount and severity, concomitant diseases, and many more). Unrealistic worries such as those about airborne transmission of peanut particles or severe reactions from skin contact should be taken away from patients. Patients should be encouraged in their ability to effectively treat allergic reactions with available emergency medications. In this way, an unduly severe reduction in quality of life can be avoided.

In the absence of a curative therapy, peanut allergy represents a lifelong burden for most patients. Considerations for the development of hypoallergenic foods [68] have not yet found their way into everyday practice. Recent investigations with allergen immunotherapy for the treatment of peanut allergy have been performed, with the aim of increasing patients’ tolerability threshold. By desensitizing patients, the amount of peanut needed to trigger a reaction increases, and the possibility of patients reacting when accidentally exposed to peanut is thereby reduced.

The approach more widely studied in clinical trials and advanced in terms of clinical experience is oral immunotherapy (OIT), which undoubtedly reduces the likelihood of reacting to peanuts. A preparation for OIT to mitigate allergic reactions after accidental exposure to peanuts in individuals aged 4–17 years with a confirmed diagnosis of peanut allergy, “defatted powder of *Arachis hypogaea* L., semen (peanuts)”; (previously known as AR101), was approved in December 2020. This is discussed in detail in the article by Blümchen et al. in Part 3 of this white paper on peanut allergy (Blümchen et al. in this issue of *Allergo Journal*).

It has been demonstrated that OIT for peanut allergy is efficacious and has a manageable safety profile with few severe adverse reactions (Blümchen et al. in this issue of *Allergo Journal*). Positive health economic outcomes can be achieved with OIT, but most importantly, quality of life improves in patients undergoing OIT, even in those not achieving sustained immune tolerance. If, however, the baseline quality of life is not impacted, it is possible that the regimented treatment and side effects may lead to a deterioration of the patient’s quality of life while on treatment. Therefore, prior to treatment, a detailed discussion on the benefits and risks of immunotherapy, taking into account all specificities of each patient and family, is desirable. Additional data are needed to better understand the longer-term profile of the treatment and to answer questions such as which patients continue to be at risk of anaphylaxis and who must continue to practice avoidance of peanuts and carry an emergency kit.

In any case, good and qualified nutritional counselling with regard to the recognition of risk situations and, at present, training in the safe use of the emergency kit for each patient remain the central components of therapy.

## Abbreviations

AGATE: Arbeitsgemeinschaft Anaphylaxie—Training und Edukation e. V.
Ara h: Allergens in peanuts (*Arachis hypogaea*)
DAAB: Deutscher Allergie- und Asthmabund (German Allergy and Asthma Association)
DBPCFC: Double-blind placebo-controlled food challenge
IgE: Immunoglobulin E
LMIV: Lebensmittel-Informationsverordnung (European Food Information Regulation)
OIT: Oral immunotherapy
